# Phytochemical Analysis, Antimicrobial Screening and In Vitro Pharmacological Activity of *Artemisia vestita* Leaf Extract

**DOI:** 10.3390/molecules29081829

**Published:** 2024-04-17

**Authors:** Shivani Dogra, Bhupendra Koul, Joginder Singh, Meerambika Mishra, Dhananjay Yadav

**Affiliations:** 1 Department of Microbiology, Lovely Professional University, Phagwara 144411, Punjab, India; shvndogra241995@gmail.com; 2 Department of Biotechnology, Lovely Professional University, Phagwara 144411, Punjab, India; 3 Department of Botany, Nagaland University, Zunheboto 798627, Zunheboto, India; joginder@nagalanduniversity.ac.in; 4 Department of Infectious Disease and Immunology, College of Veterinary Medicine, University of Florida, Gainesville, FL 32611, USA; meerambika.mishra@gmail.com; 5 Department of Life Sciences, Yeungnam University, Gyeongsan 38541, Republic of Korea

**Keywords:** *Artemisia vestita*, antimicrobial, cytotoxicity, anti-inflammatory, antioxidant, wound healing

## Abstract

*Artemisia vestita* Wall. Ex Besser is a folklore medicinal plant that belongs to Asteraceae family and a treasure trove of drugs. The aim of this research study was to investigate the phytoconstituents, antimicrobial activity, antioxidant, anti-inflammatory, cytotoxicity and wound healing potential of *A. vestita* leaf extract (ALE). Phytochemical analysis of the ALE was carried out by Soxhlet extraction and GCMS (gas chromatography–mass spectrometry) analysis. Antimicrobial activity was performed by the agar well diffusion method against selected bacterial and fungal strains. Free radical scavenging potential was evaluated by DPPH (2,2-Diphenyl-1-picrylhydrazyl), ABTS (2,2′-azino-bis(3-ethylbenzothiazoline-6-sulfonic acid)) and FRAP (Ferric reducing antioxidant power) assays. Anti-inflammatory activity was performed by enzyme inhibition assay–COXII. The cytotoxicity of ALE on HaCaT cells was studied via MTT (3-[4,5-dimethylthiazol-2-yl]-2,5 diphenyl tetrazolium bromide) assay. An in vitro scratch assay was performed for the evaluation of the wound healing property of ALE. It showed satisfactory antimicrobial activity against *Staphylococcus aureus* (14.2 ± 0.28 mm), *Escherichia coli* (17.6 ± 0.52 mm), *Bacillus subtilis* (13.1 ± 0.37 mm), *Streptococcus pyogenes* (17.3 ± 0.64 mm), *Proteus mirabilis* (9.4 ± 0.56 mm), *Aspergillus niger* (12.7 ± 0.53 mm), *Aspergilus flavus* (15.3 ± 0.25 mm) and *Candida albicans* (17.6 ± 0.11 mm). In ALE, 36 phytochemicals were detected by GCMS analysis, but 22 were dominant. Moreover, the ALE was effective in scavenging free radicals with different assays and exhibited reasonable anti-inflammatory activity. The MTT assay revealed that ALE had a cytotoxic effect on the HaCaT cells. The scratch assay showed 94.6% wound closure (after 24 h incubation) compared to the positive control Cipladine, which is remarkable wound healing activity. This is the first report on the wound healing property of *A. vestita*, which can serve as a potential agent for wound healing and extends knowledge on its therapeutic potential.

## 1. Introduction

Proper care of wounds is an important aspect in promoting rapid regeneration of skin, early healing and prevention of infections. It involves proper management. Folklore medicines are our native heritage. They have been used since time immemorial for maintaining a healthy life as mentioned in Unani, Ayurveda, Homeopathy, Traditional Chinese medicine and other traditional medicinal regimes [1,2,3,4]. These medicines are derived from traditional herbs by taking clues from their traditional uses. Novel potential bioactive compounds can be extracted, drug formulations can be prepared and more evidence regarding their efficacy can be accumulated through reverse pharmacology and ethnopharmacology. Due to growing interest from scientists and pharmacologists in the genus Artemisia, many species have been heavily explored. It is all because of their rich phytochemical compositions and therefore herbal formulation of *A. abrotanum* L., *A. absinthium* L., *A. afra*, *A. annua* L., *A. capillaries Thunb.*, *A. maritima*, *A. herba-alba*, *A. indica Wild.*, *A. japonica Thunb.*, and *A. vulgaris* have been commercialized [5,6]. One such species which is less explored but gaining much attention is *Artemisia vestita* Wall. ex Besser. It is distributed in East Asia including the mid-hill region of the Himalayas of India, Nepal, Tibet and Pakistan, at an elevation of 1500–3500 m above sea level [7]. It is a highly enriched traditional medicinal plant popularly known as “Russian Wormwood” and “Kubsha” that belongs to the Asteraceae family and is capable of treating wounds, infections, heat rashes and itching due to its cold nature [8]. *A. vestita* is a perennial, erect shrub that attains a height of 5–120 cm. Organoleptic characters include soft and hairy (hairy on upper and lower side) fern-like silvery-green foliage, with a strong smell and a bitter flavour. The flowers are hermaphrodite, small, creamy yellow in color and arranged in 6–10 racemes. The flower heads are hairy, long and compound, and gracefully hang on their slender nodding stalks. Bracts are membranous and oblong, hermaphrodite with a characteristic sweet, woody odour, and bitter in taste. 

The phytoconstituents reported in *A. vestita* belong to the chemical classes of mono and di-terpene hydrocarbons, oxygenated monoterpenes, triterpenes, sesquiterpenoids, flavonoids, alkaloids, carotenoids, monoterpenoids, phenylpropanoids, phenolic compounds, azulenes, acetylenes, aromatic aldehydes, coumarins, tannins, hydroxyl cinnamic acids, organosulfonic acids and sterols [9,10,11,12,13]. Oxygenated monoterpenes are responsible for its antimicrobial activity, phenolic compounds for its antioxidant activity, and flavonoids for its anti-inflammatory and cytotoxic activity [14,15,16]. Oxygenated monoterpenes are effective against microbes, especially in wound infections caused by bacteria and fungi [17]. Initial inflammation is a protective response against allergens, toxins and microbes, but chronic inflammation is detrimental to tissues [18]. Various phytoconstituents found in the *Artemisia* species have been reported to exhibit anti-inflammatory activity by downregulating the expression of protein-coding genes. Quercetin and kaempferol inhibit COX-II, iNOS and CRP (C-reactive protein) and downregulate TNF-α and NF-κB secretion; luteolin inhibits IL-6, IL-1β and TNFα secretion; whereas naringenin downregulates the secretions of proinflammatory cytokines, namely, TNFα, IL-6, IL-8 and IL-1β, and inhibit NF-κB activation and iNOS expression [19,20,21]. 

The antioxidant property of phytochemicals is usually estimated by using the combination of several assays because of diversity in their modes of action. Antioxidant assays can be categorized into two types depending on the chemical reactions: (i) assays which involve the single transfer of electrons based on redox reactions such as DPPH, FRAP, ABTS and NO scavenging assays, and (ii) ORAC assay which is based on transfer of hydrogen atoms [22,23]. The root cause of progression and development of various diseases is oxidative stress. Antioxidants scavenge free radicals from our bodies and play a vital role in boosting health by preventing or reducing damage caused by oxidation [24,25]. Indigenous knowledge regarding the wound healing property of the *A. vestita* leaf has been used by practitioners and local people of Himachal Pradesh (H.P.) for decades. 

The fresh leaf paste of *A. vestita* has been used as a source of medicine on wounds for speedy recovery. Thus, the wound healing property of this magical herb has been time-tested, but this traditional knowledge must be backed by scientific evidence. However, to date there have been fragmentary research reports on the wound-healing property of *A. vestita* and no specific article has been published exploring its wound healing, antimicrobial, anti-inflammatory and cytotoxic activity using leaf extract. We therefore intend to bridge the gap, and through this research we have tried to explore the in vitro pharmacological potential of Russian wormwood. The main aim of our research was phytochemical analysis, antimicrobial screening and in vitro pharmacological activity of *A. vestita* leaf extract (ALE), so as to commercialize it as a cost-effective herbal formulation for treating wound and dermatological infections.

## 2. Results and Discussion

### 2.1. Preliminary Screening

10.95 g of crude extract was obtained from 25 g of leaf powder through soxhlet extraction. Preliminary phytochemical analysis of the crude extract confirmed the presence of flavonoids, terpenoids, alkaloids, glycosides, tannins and phenolic compounds (Table 1).

### 2.2. Phytochemical Analysis through GCMS

Phytochemicals are chemical compounds which form during metabolic processes of the plants, also known as secondary metabolites. Here, the aim was to screen the main chemical composition of ALE by GCMS. A total of 22 compounds were identified accounting for 94.89% of the total composition which showed various peaks. They were identified as grandisol, 1,8-cineole, camphor, limonene, β-caryophyllene, thujone, isofraxidin, myrcene, naringenin, camphene, santolina triene, apigenin, α-pinene, yomogin, germacrene-D, artemisia alcohol, borneol, cirsilineol, quercetin, artemisia ketone, copaene and amyrin (Supplementary Figure S1) after comparison with the extant literature, mass spectra and retention time stored in the NIST library (http://www.nist.gov/srd/nist1a.cfm, accessed on 18 July 2022). The molecular weight, percentage area, molecular formula, retention time, structure and biological activity of the identified compounds are summarized in Supplementary Table S1. 

As per review of the literature, a total of 202 phytochemicals have been reported from whole-plant (stem, roots and leaves) essential oil which belongs to different classes [8,12,26,27]. It has been reported that *A. vestita* essential oil composition is highly influenced by climatic conditions and geographical region. Phytochemical studies showed *A. vestita* contains camphor, eucalyptol and thujone which are predominantly present in other species of Artemisia [24,28,29]. Oil odour is determined by the presence of thujone, atlantone, 1,8-cineol and himachalol compounds. Oxygenated monoterpenes are known for their antimicrobial activity. β-caryophyllene, artemisia ketone, 1,8-cineol, artemisia alcohol and α-phellandrene were major active compounds of essential oil reported from Nainital (29.3924° N, 79.4534° E), Uttarakhand, whereas camphene, α-pinene, artemiseole, α-myrcene, 1,8-Cineol (Eucalyptol), camphor, pinocamphone, δ-terpinene, a-pinocarvone, grandisol, terpinen-4-ol, γ-pyronene, β-cubene, copaene, α-caryophyllene, α-amorphene, γ-himachalene, germacrene D, caryophyllene, aromadendrene compounds were found from Mentougou District (39.9397° N, 116.1014° E), Beijing. Apigenin, pectolinarigenin, cirsiliol, 7-methoxycoumarin, annphenone, umbelliferone, scopoletin, quercetin, cirsilineol, jaceosidin, cirsimaritin, hispidulin, 6-methoxytricin and acacetin were found from Lhasa (29.6526° N, 91.1378° E), Tibet. Scopoletin, luteolin, rutin, naringenin, eugenol, kaempferol, isoeugenol, artemether, himachalene, germacrene-D, caryophyllene, allohimachalol, himachalol, atlantone, yomogi alcohol, santolina alcohol and thujanols were found from Srinagar (34.0837° N, 74.7973° E), Kashmir. α-Amyrin, stigmasterol, β-sitosterol, scopolin, umbelliferone, daucosterol, arvestolides D–J, arvestonol and arvestonates A–C were major active compounds from China (35.8617° N, 104.1954° E) [9,15,21,30,31,32].

### 2.3. Antimicrobial Activity

The antimicrobial activity of ALE was evaluated against microbial strains causing skin infections by using the agar well diffusion method. These included *S. pyogenes*, *S. aureus*, *E. coli*, *B. subtilis*, *P. mirabilis*, *A. flavus*, *A. niger* and *C. albicans*. The potency of the plant extract was assessed by zone of inhibition (ZOI) against the standard drug azithromycin for bacteria and fluconazole for fungi, and further minimum inhibitory concentration (MIC) and minimum bactericidal concentration (MBC) values were determined. The results are presented in Table 2, which indicates plant extract exhibiting antimicrobial activity against different microbial strains that cause skin infections. It was interesting to know that ALE showed satisfactory results as compared to synthetic drugs. *A. vestita* extract exhibited high antimicrobial activity against *E. coli*, *S. pyogenes* and *C. albicans* as indicated by the maximum ZOI: 17.6, 17.3, 17.6 mm, respectively, whereas intermediate ZOI: 14.2, 13.1, 15.3, 12.7 mm were observed against *S. aureus*, *B. subtilis*, *A. niger* and *A. flavus*, respectively. *P. mirabilis* showed the least ZOI (9.4 mm). Results of the zone of inhibition are present in Table 2.

The decreasing order of the antibacterial activity based on ZOI values was as follows: *E. coli* (17.6 mm) > *S. pyogenes* (17.3 mm) > *S. aureus* (14.2 mm) > *B. subtilis* (13.1 mm) > *P. mirabilis* (9.4 mm) and antifungal activity was: *C. albicans* (17.6 mm) > *A. flavus* (15.3 mm) > *A. niger* (12.7 mm). The MIC/MBC/MFC value of bacteria ranges from 100 to 250 µg/mL, whereas for fungi, the value ranges from 100 to 300 µg/mL, respectively, which is satisfactory compared to the standard drug azithromycin and fluconazole. These results were similar to those of a previous published report on essential oil versus grandisol, the latter is major constituent of *A. vestita*, offering potential as a novel drug against *S. pyogenes* for treatment of respiratory infections which exhibit a MIC/MBC range between 130 and 200 µg/mL [33]. Components that contributed to antibacterial activity were α-pinene, thujone, nerol, 1,8-cineole, terpineol and oxygenated monoterpenes [34]. The results indicated that *A. vestita* extract has potent antimicrobial effects; can cure various dermal infections like acne, boils, wound infections, etc., caused by both bacteria and fungi; and could be a promising antimicrobial drug candidate. These results encouraged us to conduct experiments on in vitro wound healing, antioxidant, anti-inflammatory and cytotoxic activity of *A. vestita* leaf extract.

### 2.4. Antioxidant Activity

**Figure 1:**
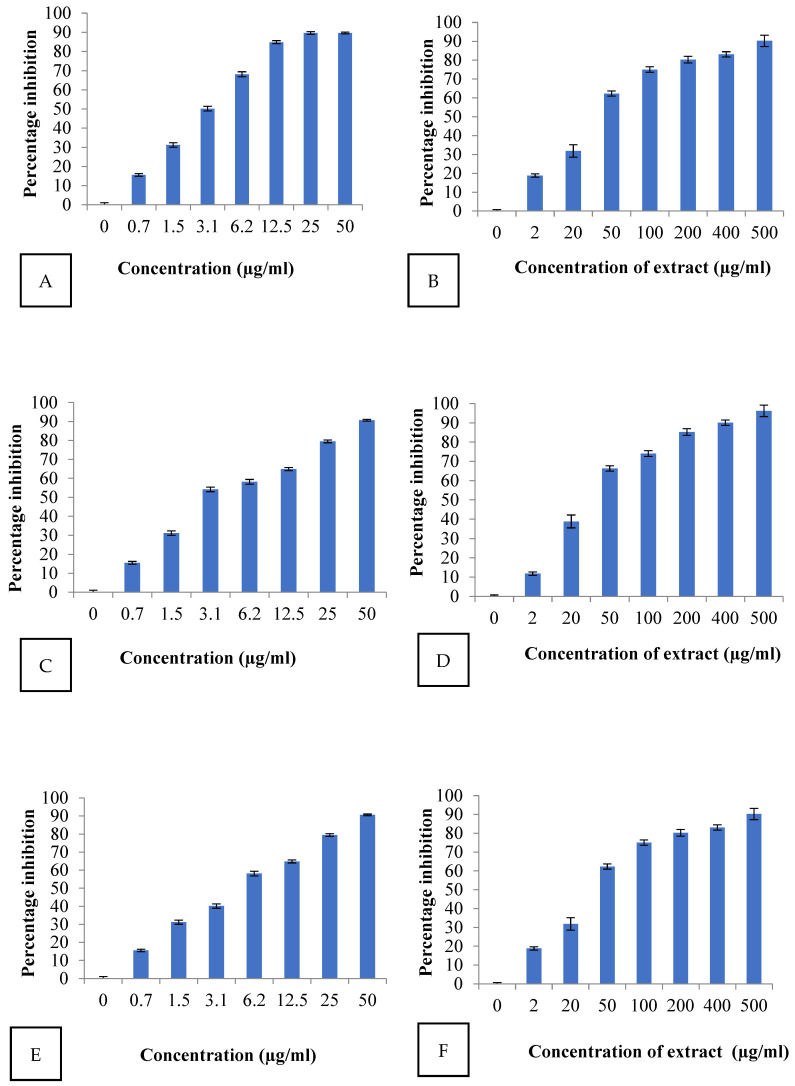
Scavenging activity of ascorbic acid and *Artemisia vestita* leaf extract (ALE) on (**A**,**B**) DPPH (IC50 value: 3.11 µg/mL and 32.474 µg/mL), (**C**,**D**) FRAP (IC50 value: 2.572 µg/mL and 39.11 µg/mL) and (**E**,**F**) ABTS (IC50 value: 6.085 µg/mL and 28.82 µg/mL), respectively.

DPPH scavenging activity was performed with ALE. IC50 is a half maximal inhibitory concentration used to analyze the antioxidant potential of a sample. The lower the IC50 value, the higher the antioxidant potential. In this study, IC50 was calculated by using the laboratory tool IC50/IC9 calculation module. It was observed that ALE is a more active DPPH radical scavenger than ascorbic acid (positive control). Results of DPPH free radical scavenging activity, ABTS and FRAP are shown in Figure 1 and Table 3.

The antioxidant activity (based on IC50 values) trend with ALE was observed as: FRAP > DPPH > ABTS. ALE exhibited significant antioxidant activity on DPPH free radicals with a IC50 value of 32.474 µg/mL with respect to standard ascorbic acid, which shows it was able to reduce 50% stable DPPH radical (violet) to DPPH-H (yellow). The IC50 value (28.82 µg/mL) obtained in the ABTS assay was lower than that of DPPH, whereas ALE was able to reduce the 50% ferric ions to ferrous (Fe^2+^) ions with a IC50 value of 39.11 µg/mL. The results are in agreement with the findings on other species of Artemisia which also exhibited the same trend FRAP > DPPH > ABTS [35].

### 2.5. Enzyme Inhibition Assay- Cox-II

**Figure 2:**
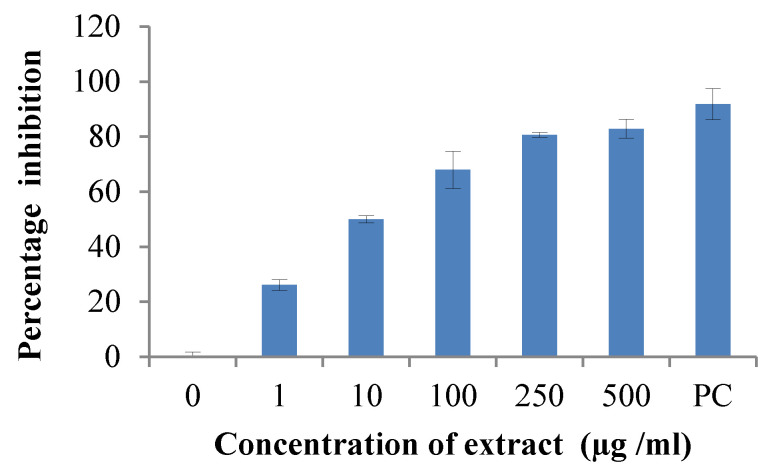
Inhibition of COX-II enzyme at different concentrations of *Artemisia vestita* leaf extract (ALE).

ALE exhibited significant anti-inflammatory activity with a IC50 value of 11.46 µg/mL, as represented in Figure 2, and may be useful for nutritional and therapeutic purposes.

Flavones such as apigenin, cirsilineol and 6-methoxytricin from *A. vestita* have shown immunosuppressive and anti-inflammatory effects [11]. The Artemisia species are widely used in China and Tibet for treating various inflammatory diseases such as sepsis, contact dermatitis and rheumatoid arthritis [36,37,38].

### 2.6. Cytotoxic Effect of Artemisia vestita Extract

**Figure 3:**
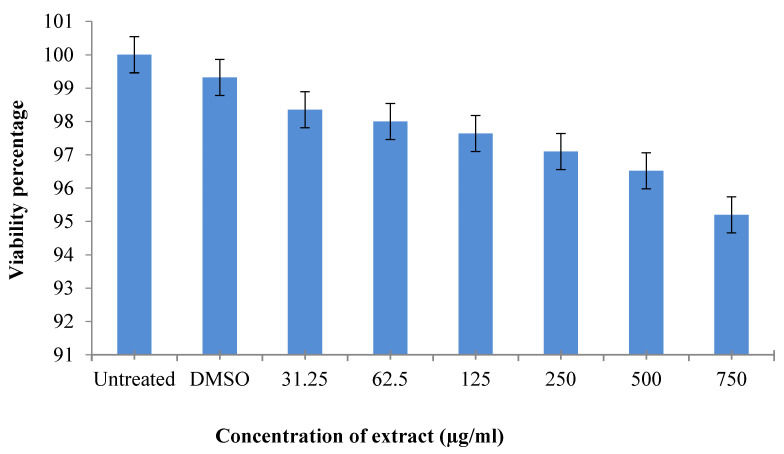
The cytotoxic effect of *A. vestita* leaf extract (ALE) on HaCat cells.

An MTT assay was performed for the evaluation of ALE cytotoxicity against theHaCat cell line. In Figure 3, the bars represent the percent viability of HaCaT cells, which was observed at different concentrations of ALE (31.25, 62.5, 125, 250, 500, 750 μg/mL) after an incubation of 24 h, as represented in Table 4. It was observed that ALE was a mild cytotoxic and can be used as novel complementary therapy with certain modifications against cancers. 

The results were in consonance with those of other species such as *A. fragrans*, *A. incana* and *A. spicigera* [39]. They did not show cytotoxic effects against MCF7 and HEK293 cells. But researchers have hypothesized that *A. absinthium* and *A. vulgaris* might have anticancer compounds as they showed mild cytotoxic effects against MCF7 and HEK293 cells [40,41,42,43]. Some researchers reported the presence of volatile monoterpenes and terpenoids such as citronellol, limonene, terpinolene, thymol, camphor, pinene and thymol, and that they show repellent or cytotoxic activity [44].

### 2.7. Wound Healing Activity

**Figure 4:**
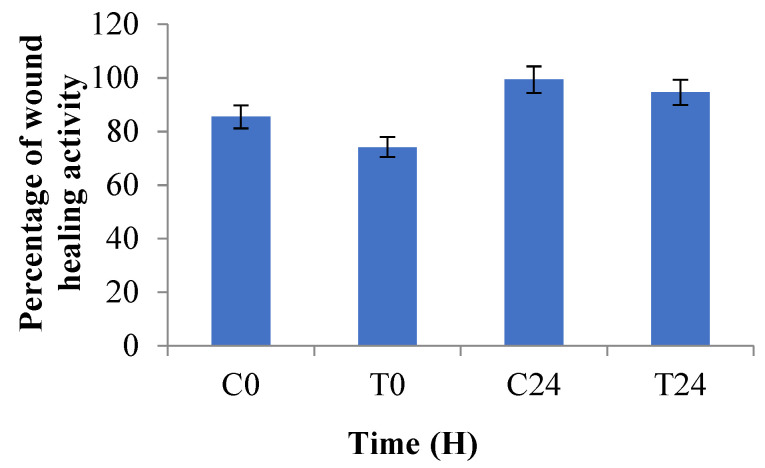
Comparative analysis of wound healing activity of cipladine (positive control) and *Artemisia vestita* leaf extract at 0 and 24 h. C0—Control (Cipladine) at 0 h; C24—Control (Cipladine) at 24 h; T0—Treatment *(Artemisia vestita* leaf extract) at 0 h; T24—Treatment (*Artemisia vestita* leaf extract) at 24 h.

**Figure 5:**
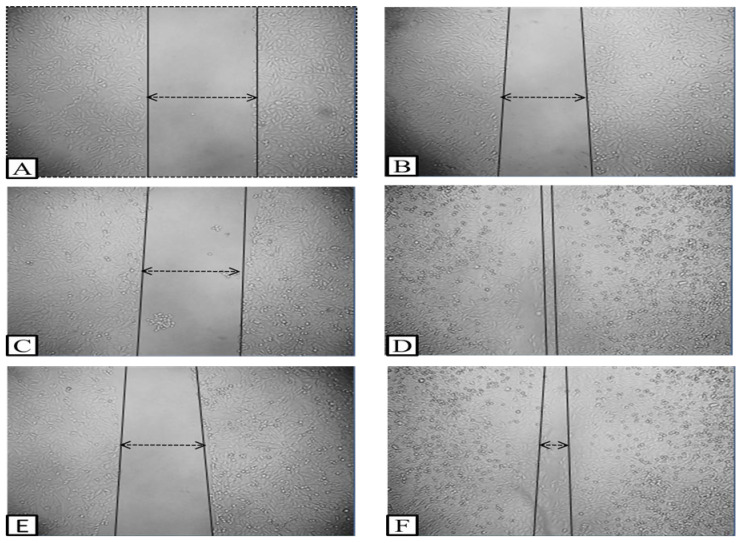
Microscopical images representing the nature of in vitro wound healing. (**A**,**B**) No treatment; (**C**,**D**) Cipladine (positive control); (**E**,**F**) *Artemisia vestita* extract treatment at 0 and 24 h, respectively. 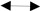: represents wound closure in images.

These days, scratch assays widely follow the in vitro technique for better understanding of medicinally important phytoconstituents. In this research, HaCaT cells were treated for 24 h with 125 μg/mL of ALE. Cell migration was captured at 0 and 24 h and the distance of wound closure was calculated by the software Image J version 1.54 (NCBI). The results indicated that ALE at concentration 125 μg/mL closed the gap by 94.625% in 24 h. Percentage of wound closure with respect to 0 and 24 h in cipladine treated cells and ALE-treated cells are represented in Figure 4. In Figure 5, microscopical images represent the nature of in vitro wound healing: (A, B) untreated cells with no change; (C, D) Cipladine (positive control); and (E, F) *Artemisia vestita* extract treatment at 0 and 24 h, respectively.

Here, Figure 5 shows that with ALE treatment, wounds achieve closure (94%) (image F) in comparison with the standard drug Cipladine (99.05%) (image D) within 24 h which showed satisfactory results. A previously published review also stated that Chinese medicines including *A. vestita* serve as an alternate or complementary medicine for therapeutic purposes such as application on cuts and wounds to stop inflammation and bleeding [45]. Flavonoids play an important role in wound healing. Other species of this genera such as *A. scoparia Waldst. Kit* and *A. parviflora* are also used for wound healing purposes. Decoction from these species has been formulated and traditionally used in Pakistan to cure skin infections, injury and wounds [46,47].

As per the literature, many reports are affirming the various activities of the plant genera Artemisia with its essential oil but so far, there are no reports on the crude extract of *Artemisia vestita* showing its antimicrobial, antioxidant, anti-inflammatory, cytotoxicity and wound healing activity. As aforementioned, the leaf paste is used as traditional medicine generally for wound healing, but to increase its shelf life and related efficacy, ALE needs to be modified into a gel. Patents that have been published from the work reported in this manuscript are as follows:

- “A novel composition of hydrogel containing *Artemisia vestita* and process thereof” (Application no.—202311069887).
- “Enhancement of the wound healing capability of *Artemisia vestita* leaf extract using biodegradable Tragacanth gum-based hydrogel” (Application no.—202311087841).

## 3. Materials and Methods

### 3.1. Selection and Identification of Plant Material

**Figure 6:**
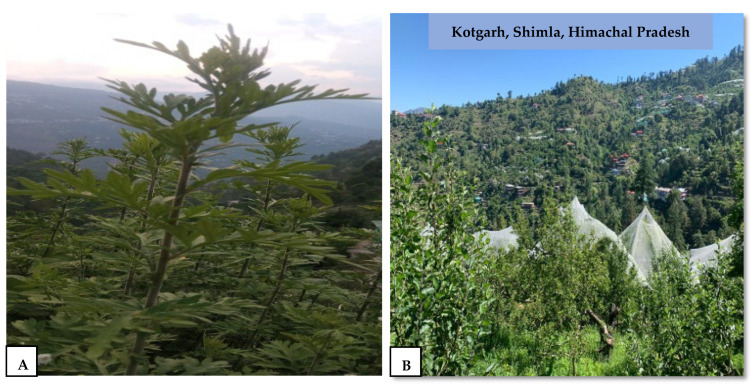
*Artemisia vestita* plants: (**A**) habitat and (**B**) geographical location (Kotgarh, Shimla, HP) of the study area.

The fresh leaves of *Artemisia vestita* were collected in the months of April–May at the flowering stage from the wild forest of Kotgarh 31.31° N 77.47° E situated in district Shimla, Himachal Pradesh, India (Figure 6) and were further stored and shade-dried. The plant authentication was performed in Dr. Yashwant Singh Parmar University of Horticulture and Forestry, Solan (HP) (UHF-Herbarium no. 13916).

### 3.2. Plant Extract Preparation

The plants were shade-dried for 15 days and their leaves were crushed to a coarse powder using a mechanical grinder. The powder was further passed through a fine mesh and stored at room temperature in a clean glass jar until further use. A total of 25 g of coarse powder was weighed and filled in the filter paper thimble in Soxhlet apparatus (250 mL capacity) and the process was carried out as per standard protocol [48]. Parts of apparatus include the condenser, thimble and round bottom flask, the last of which is kept on the heating mantle. The sterile double-distilled water (solvent) was made to run down 2–3 times through the thimble to the round bottom flask. The temperature was set at 55 °C and was maintained till extraction was complete. The extract from the flask was concentrated to a semisolid form (10.95 g) at 40 °C for 15 min in a hot air oven and stored at 4 °C for later use.

$$\%\;\mathrm{of}\;\mathrm{extract}\;=\frac{\mathrm{Weight}\;\mathrm{of}\;\mathrm{concentrated}\;\mathrm{extract}}{\mathrm{Weight}\;\mathrm{of}\;\mathrm{plant}\;\mathrm{sample}}\times 100$$

The semisolid extract was suspended in 100 mL of double-distilled water to make a stock of 100 mg/mL. For the MTT assay and in vitro scratch assay, the stock was prepared in 0.5% DMSO.

### 3.3. Preliminary Screening of Phytochemical

Preliminary Screening was performed to determine the presence or absence of different phytochemical compounds present in the ALE, viz., alkaloids (Dragendorff’s test), flavonoids (Shinoda test), terpenoids (Salkowski test), tannins and phenolic compounds (5% FeCl_3_ test) [49].

1. Test for phenolic compounds—To 2 mL of ALE, a few drops of 5% FeCl_3_ was added in a test tube. A deep blue-black colour indicated the presence of phenolic compounds.
2. Shinoda test—To 1 mL of ALE in a test tube, 10% lead acetate solution was added. A yellow colour precipitate indicated the presence of flavonoids.
3. Dragendorff’s test—To 2 mL of ALE in a test tube, a few drops of Dragendorff’s reagent were added. A orange-brown precipitate indicated the presence of alkaloids.
4. Test for tannins—To 5 mL of bromine water in a test tube, 1 mL of ALE was added. Discoloration in solution showed the presence of tannins.
5. Salkowski test—To 2 mL of ALE in a test tube, 2 mL of chloroform was added. Development of a reddish-brown layer after the addition of 1 mL H_2_SO_4_ indicated the presence of terpenoids.

### 3.4. GC-MS (*Gas Chromatography and Mass Spectrometry)*

The ALE was also subjected to GC-MS which was performed on Agilent 7010B GC/TQ, fitted with a fused silica column VF-5 MS (60 × 0.25 mm; film thick 0.25 μm) split less injection, which was coupled with a Mass Detector Series 400 (Agilent technology, Santa Clara, CA, USA) under the following conditions: constant flow of carrier gas helium at 1 mL/min; injection volume 1:80 (0.8 μL); injector temperature maintained at a constant 250°C. The column oven temperature was set initially at 40 °C for 1 min, raised 10 °C/minute up to 150 °C and the final temperature was increased to 260 °C for 10 min. For the detection of GCMS spectra, high ionization energy of 70 electron volt with an ion source temperature of 260 °C was used, and the MS were recorded between the range of 50 and 500 amu. Identification of the phytochemicals were based on experimental retention index (RI), observed with respect to homologous n-alkenes series (C8–C40) under the same experimental conditions: further co-injection with the standards and identified using the MS NIST library available online (software version: 2.4; data version: NIST v20) (http://www.nist.gov/srd/nist1a.cfm, accessed on 12 April 2022). The constituents relative percentages were calculated from the peak areas obtained from the chromatogram.

### 3.5. Antimicrobial Activity

The bacterial cultures of *Bacillus subtilis* (MTCC 2395), *Staphylococcus aureus* (MTCC 3160), *Escherichia coli* (MTCC 443), *Streptococcus pyogenes* (MTCC 1927), *Proteus mirabilis* (MTCC 425), *Aspergillus niger* (MTCC 281), *Aspergillus flavus* (MTCC 9390) and *Candida albicans* (MTCC 183) used in the current study were procured from the Microbial Type Culture Collection and Gene Bank, Institute of Microbial Technology (IMTECH), Chandigarh. The microbial strains were cultured overnight before conducting the antimicrobial activity experiments. The microbial cell suspension in 0.5% sodium chloride (NaCl) were adjusted to standard 0.5 McFarland to obtain approximately 106 CFUs/mL (colony forming units).

#### 3.5.1. Agar Well Diffusion Method

The agar well diffusion method was performed to check the antimicrobial activity of ALE. Nutrient agar (Sigma Aldrich, St. Louis, MO, USA) media and potato dextrose agar were autoclaved and poured into the Petri plates under laminar air flow and then kept overnight for solidification. The next day, the overnight bacterial culture (500 μL) was uniformly spread on the solidified nutrient agar with the help of an L-shaped spreader. Thereafter, 6 mm diameter wells were made using a cork borer.A small fraction (100 μL) of drug (25, 50, 100 µg/mL) was filled in the first 3 wells, respectively, and a positive control (10 µg/mL) azithromycin for bacteria and fluconazole for fungi was taken in the last well. The Petri plates were incubated for 24 h at 37 °C for bacteria and 28 °C for fungi. Antimicrobial activity of the ALE was analysed by zone of inhibition (ZOI) expressed in mm. Experiments were performed in triplicate.

#### 3.5.2. Minimum Inhibitory Concentration (MIC) and Minimum Bactericidal Concentration (MBC)

The MIC of the ALE was determined by the serial dilution method (CLSI protocol), as described by Eloff (1998) [50]. Nutrient broth (Hi Media, Mumbai, India) was used for the culture of *S. aureus*, *B. subtilis*, *S. pyogenes*, *E. coli* and *P. mirabilis*, while potato dextrose broth (Hi Media, India) was used for the culturing of *A. flavus*, *A. niger* and *C. albicans*. In first phase, the ALE concentration was taken as 10, 20, 30 and 40 µg/mL. There was no such inhibition of bacteria and fungi. In the second phase, the drug concentration was taken as 100, 150, 200, 250 and 300 µg/mL. A negative control devoid of ALE was also set up to demonstrate adequate bacterial/fungal growth over the course of a particular period of incubation and media sterility, respectively. The dilutions were incubated for 24 h and were followed by observation. Turbidity indicates the growth of microbes and MIC is the lowest concentration of drug where no turbidity (no growth) is visually observed. Experiments were performed in triplicate.

### 3.6. Cell Culture

The human keratinocyte cell line (HaCaT) was procured from NCCS (National centre for cell science), Pune (India) and the cultures were maintained at Lovely Professional University, Punjab, India. The cells were grown in DMEM (Dulbecco’s modified eagle medium) (Gibco, Waltham, MA, USA), which was supplemented with 10% FBS (fetal bovine serum) and 1% antibiotics (i.e., penicillin 100 U/mL and streptomycin 100 U/mL). The cells were incubated at 37 °C with 5% CO_2_ and cells were passaged after they reached 70–80% confluency.

### 3.7. Antioxidant Activity

#### 3.7.1. DPPH Free Radical Scavenging Activity

The antioxidant activity of ALE was determined by using the free radical DPPH (2,2-diphenyl-1-picrylhydrazyl). It is an electron transfer-based free radical scavenging assay with some modifications in a previously described method [51,52]. The reagent used was 0.2 mM DPPH in ethanol. A total of 10 μL of different stock of the ALE (0–500 μg/mL) was added to DPPH solution (0.2 mL) in a 96-well plate and incubated for 30 min in the dark. After incubation, the decolorization was recorded at 495 nm spectrophotometrically using a microplate reader (iMark, Bio-Rad, Hercules, CA, USA). The reaction mixture containing deionized water (20 μL) served as control. The scavenging activity was represented as %age inhibition with respect to the control. The reaction was set in triplicate. Ascorbic acid (vitamin C) served as the standard and DPPH solution was taken as the blank.

Calculations are as follows:

DPPH scavenging activity (%) = (Abs_Control_ − Abs_Sample_)/Abs_Control_) × 100

where, Abs_Control_ = absorbance of the control; Abs_Sample_ = absorbance of the sample.

The lower the IC50 value, the greater the antioxidant activity is. The %age of inhibition was plotted against concentration, and the line obtained was used to obtain the IC50 value.

#### 3.7.2. ABTS Radical Scavenging Assay

The 2,2-azinobis,3-ethyl-benzothiazolin-6-sulfonic acid (ABTS) principle is based on the generation of ABTS radicals. It forms a blue-green complex when oxidized with potassium persulphate [53] in the dark for 12–18 h at 4 °C. A total of 2 mL of ABTS solution (0.192 g in 50 mL of methanol) was added to different concentrations of ALE (0–500 μg/mL) and incubated at room temperature. Observations were recorded spectrophotometrically at 745 nm using a micro plate reader (iMark, Bio-Rad, USA). Ascorbic acid was used as the standard. The percentage of inhibition was calculated by using the following formula:

Scavenging activity (%) = (Abs_Control_ − Abs_Sample_)/(Abs_Control_) × 100

where, Abs_Control_ = absorbance of the control, and Abs_Sample_ = absorbance of thesample.

#### 3.7.3. Ferric Reducing Antioxidant Power (FRAP) Assay

The ferric reducing antioxidant power (FRAP) assay is based on the reduction of ferric tripyridyltriazine to ferrous tripyridyltriazine by the potent antioxidant present in sample. It develops a blue hue in the presence of cyanide ions and ferric ions. The ALE reducing power was determined by slight modification in the method described by Yen and Duh (1994) [54]. On the day of analysis, 2.4 mL of FRAP reagent (25 mL buffer + 2.5 mL TPTZ and FeCl_3_·6H_2_O) was added to ALE (0–500 μg/mL) at different concentrations and then further incubated in the dark at room temperature for 30 min. The observation was recorded spectrophotometrically at 593 nm using a microplate reader (iMark, Bio-Rad, USA). Reduction ability increases with the increase in absorbance. Ascorbic acid (Merck, India) was used as the control while distilled water was used as a blank. The lower the value of RP50 (reducing power), the greater the reducing power ability.

% Reducing power (RP) = (Abs_Control_ − Abs_Sample_)/(Abs_Control_) × 100

### 3.8. Enzyme Inhibition Assay- Cox-II

The ALE was diluted at different concentrations (0–500 μg/mL) in buffer (Tris HCl buffer, pH 8.0) and prepared as described by Gierse (1998) [55]. Reaction components were placed in each well of a 96-well plate. The enzymatic reaction was initiated by adding 5 μL substrate and 5 μL TMPD solution and then the plates were incubated for 10 min at room temperature and absorbance was read at 595 nm by using a micro plate reader (iMark, BioRad). Colexcib (17 μM) was used as the control.

### 3.9. Cytotoxic Activity

#### 3-(4,5-Dimethylthiazol-2-yl)-2,5-diphenyl Tetrazolium Bromide MTT Assay

Cytotoxicity evaluation of ALE on the HaCaT cell line was carried out according to the protocol provided by the American Type Culture Collection (ATCC) [56,57,58]. The cells (5000–8000 cells/well) were cultured in 96-well plates for 24 h in DMEM (Dulbecco’s Modified Eagle Medium) supplemented with 10% Fetal Bovine Serum and 1% antibiotic solution (Penicillin and Streptomycin) at 37 °C with 5% CO_2_. The next day, the medium was removed, and fresh culture medium was added to each well of the plate. A total of 5 µL of treatment dilutions of different concentrations, i.e., 31.25, 62.5, 125, 250, 500, and 750 µg/mL, were added to the defined wells, and treated plates were incubated for 24 h. After incubation, the medium was removed and the MTT reagent was added (50 μL of 5 mg/mL) and incubated in a CO_2_ incubator for 4 hat 37 °C. The formazan crystals were solubilised with 100 μL of (Dimethylsulfoxide) DMSO. Finally, the plates were read at 550/660 nm using a micro plate reader (iMark, BioRad). Untreated cells were control showed 100% viable.

The experiment was performed in triplicate and the %age of cell viability was plotted against the different concentrations of test samples. The HaCat cells treated with ALE were compared with the untreated cells (control). The cells which were exposed to toxins would show decreased activity. The %age of cell viability was calculated by using the below mentioned formula.

$$\%\mathrm{age}\;\mathrm{of}\;\mathrm{viability}\;=\frac{\mathrm{Mean}\;\mathrm{absorbance}\;\mathrm{of}\;\mathrm{test}\;\mathrm{sample}\;}{\mathrm{Mean}\;\mathrm{absorbance}\;\mathrm{of}\;\mathrm{control}}\times 100$$

### 3.10. In Vitro Scratch Assay

ALE wound healing capability was observed by performing in vitro (wound healing assay) scratch assays by a previously described method [59] on HaCaT cells, (10,000 cells/well) which were cultured for 24 h in DMEM medium supplemented with 10% fetal bovine serum (FBS) and 1% (penicillin and streptomycin) antibiotic solution in a 6-well plate at 37 °C with 5% CO_2_. Cells were washed with phosphate-buffered saline (PBS) after 24 h and scratched with a 200 µL tip. Further cells were treated with a specified concentration of ALE. Cipladine (positive control) was used as a standard drug, which is commonly used for wound healing purposes [60,61]. Untreated cells were considered as negative control. Photography was performed at 0 and 24 h. The area of the gap was analyzed using Image J software version 1.54 (NCBI).

### 3.11. Statistical Analysis

All the experiments were performed in triplicate, and their results were averaged and plotted with Graph pad prism software version 6.0. (Graph Pad Software, San Diego, CA, USA). Data are present in the form of mean ± SD (standard deviation).

## 4. Conclusions

Traditional medicines are a treasure trove of new drugs. To the best of our knowledge, this is the first study providing complete data on antimicrobial, antioxidant, anti-inflammatory, cytotoxicity and wound healing activity of ALE collected from Shimla (HP) India. The phytochemical composition showed that the plant contains alkaloids, flavonoids, terpenoids, tannins, glycosides and phenolic compounds which were further identified by GCMS. ALE showed potential antimicrobial effects against microbes causing dermal infections, which is quite interesting from a pharmaceutical point of view. It is also effective in scavenging free radicals which showed its antioxidant potential. The MTT assay revealed that ALE had no such cytotoxic effects on the HaCaT cells; at higher concentrations it showed mild toxic effects on cells resulting in cell death. The wound healing potential of ALE is due to the flavonoid compounds present in it, as the wound was healed (94.6%) within 24 h of incubation compared to the positive control cipladine (99.05%), which is its remarkable property. Eighty percent of the world’s population rely on herb-based medicinal products. As per practitioners and villagers, it is a folklore medicine which is generally used for wound healing by instantly applying the leaf paste on fresh wounds. The benefits of the ALE have been time-tested, but for a longer shelf life, it requires certain modifications like its modification into a gel formulation. One of the aim behind all this research and experimental setup is to provide scientific evidence for this traditional medicine. For this, we also tried to formulate a gel after conducting all the experimental work mentioned in this manuscript.

ALE can be used as a personal care product for minor cuts, redness, inflammation, stopping bleeding, wound healing and many types of microbial infections on the skin. Its formulations can be commercialized as hydrogels in personal care products for treating dermatological infections as it will be cost-effective and a safe alternative to chemicals.

## Figures and Tables

**Table 1:** Preliminary phytochemical screening.

Constituents	Tests	Observations	Result
Glycosides	Foam test	Foam was observed	+
Flavonoids	Shinoda test	Yellow color precipitate	+
Terpenoids	Salkowski test	Reddish brown coloration	+
Alkaloids	Dragendorff’s test	Orange-brown precipitate	+
Tannins and phenolic compounds	5% FeCl_3_ solution	Deep blue-black color	+
	Tannic acid test	Brown discoloration	

**Table 2:** Antimicrobial activity of *Artemisia vestita* leaf extract (ALE).

Microbes	Zone of Inhibition (mm)				MIC and MBC/MFC (µg/mL) of ALE	
	25 (µg/mL)	50 (µg/mL)	100 (µg/mL)	Standard Drug (Azithromycin/Fluconazole)	MIC (µg/mL)	MBC/MFC (µg/mL)
*S. aureus*	9.2 ± 1.53	11.24 ± 0.53	14.2 ± 0.28	18.4 ± 0.25	100	240
*E. coli*	9.4 ± 1.84	14.5 ± 0.29	17.6 ± 0.52	20.1 ± 0.51	200	250
*B. subtilis*	5.47 ± 1.17	6.47 ± 0.41	13.1 ± 0.37	16.4 ± 0.26	150	200
*S. pyogenes*	8.12 ± 0.93	12.1 ± 0.37	17.3 ± 0.64	20.1 ± 0.21	200	250
*P. mirabilis*	5.21 ± 0.87	7.28 ± 0.63	9.4 ± 0.56	15.2 ± 0.12	100	200
*A. flavus*	10.3 ± 0.22	14.2 ± 0.31	15.3 ± 0.25	16.3 ± 0.32	100	280
*A. niger*	6.3 ± 0.31	10.7 ± 0.42	12.7 ± 0.53	14.7 ± 0.16	100	250
*C. albicans*	10.2 ± 0.36	12.1 ± 0.32	17.6 ± 0.11	20.1 ± 0.25	250	300

MBC: minimum bactericidal concentration; MIC: minimum inhibitory concentration; MFC: Minimum fungicidal concentration; ALE: Artemisia leaf extract.

**Table 3:** Antioxidant activity of *Artemisia vestita* leaf extract (ALE) based on IC50 (μg/mL).

Assay	Sample	
	Ascorbic Acid (μg/mL)	ALE (μg/mL)
DPPH scavenging activity/IC50 (µg/mL) ^a^	3.11	32.474
Ferric reducing assay power/RP50 (µg/mL) ^b^	2.572	39.11
ABTS scavenging activity/IC50 (µg/mL) ^a^	6.085	28.82

^a^: Sample concentration required to inhibit the 50% radical formation. ^b^: Sample concentration required to reduce 50% ferric to ferrous ions.

**Table 4:** MTT assay using *Artemisia vestita* leaf extract (ALE).

Extract Concentrations (μg/mL)	Viability Percentage
Untreated	100 ± 9
DMSO	99.32 ± 7
31.25	98.35 ± 15
62.5	98.0 ± 14
125	97.64 ± 9
250	97.1 ± 8
500	96.52 ± 8
750	95.2 ± 9

## Data Availability

Data will be made available on request.

## Supplementary Materials

The following supporting information can be downloaded at: https://www.mdpi.com/article/10.3390/molecules29081829/s1, Figure S1: Gas chromatography mass spectrometry of *Artemisia vestita* leaf extract (ALE); Table S1: Phytochemical compounds of *Artemisia vestita* leaf extract (ALE) from HP (India) detected by using GC-MS analysis and their biological activity.
