# Supplementary material for: Phytochemical Analysis, Antimicrobial Screening and In Vitro Pharmacological Activity of Artemisia vestita Leaf Extract
Source: Molecules. 2024 Apr 17;29(8):1829. doi: 10.3390/molecules29081829 (PMC11054168; doi:10.3390/molecules29081829)
Supplement: Supplementary file 1 [file molecules-29-01829-s001.zip › molecules-2926220-supplementary.pdf]

Supplementary material

Table S1. Phytochemical compounds of *Artemisia vestita* leaf extract (ALE) from HP (India) detected by using GC-MS analysis and their biological activity.

S No.	Retention time (min)	Name of the compound	Molecular formula	Molecular weight g/mol	Peak area (%)	Structure of compounds	Biological activity
1.	4.62	Apigenin	$C_{15}H_{10}O_5$	270.237	0.35	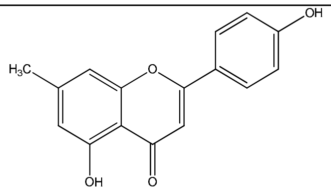	Antioxidant, antimutagenic, anticarcinogenic, anti-inflammatory, and antiproliferative activities [1,5,11]
2.	6.78	alpha-Pinene	$C_{10}H_{16}$	136.2340	8.77	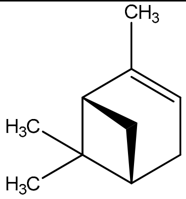	Antimicrobial, apoptotic and antibiotic [9,15,21]
3.	7.46	Camphene	$C_{10}H_{16}$	136.2340	2.29	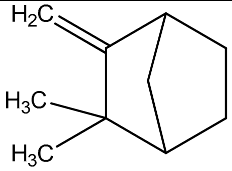	Antioxidant, antimicrobial, apoptosis [30-32]
4.	7.60	Yomogin	$C_{15}H_{16}O_3$	244.29	2.74	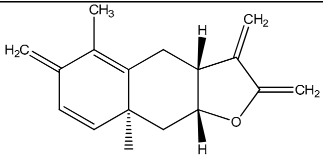	Anti-inflammatory [24,28,29]
5.	8.11	Germacrene–D	$C_{15}H_{24}$	204.3511	4.85	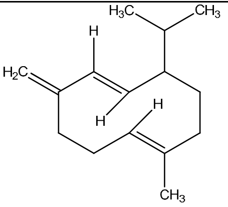	Antimicrobial [24,33,35]
6.	8.21	β-Myrcene	$C_{10}H_{16}$	136.2340	1.6	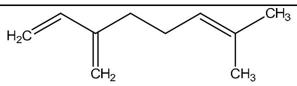	Analgesics, anti-diabetic, antioxidant, anti-inflammatory [24,28,29]

7.	8.88	Limonene	$C_{10}H_{16}$	136.23	0.26	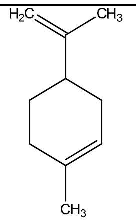	Antitumor, antiviral, anti-inflammatory, and antibacterial [24,30-32]
8.	9.33	Isofraxidin	$C_{11}H_{10}O_5$	222.1941	2.21	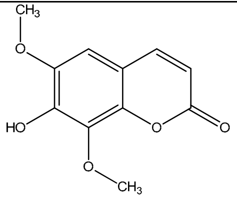	Anti-bacterial, anti-oxidant, anti-depressive, and anti-inflammatory [16,24,28,29]
9.	9.50	Camphor	$C_{10}H_{16}O$	152.2334	1.2	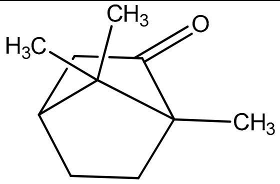	Antimicrobial, antifungal, bactericidal, and antiparasitic properties [16,30,31]
10	9.77	1,8-cineol	$C_{10}H_{18}O$	154.2493	11.35	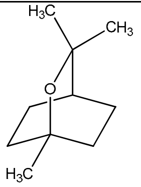	Anti-inflammatory, antioxidant, antimicrobial [1,2,5,8,14]
11	10.53	Santolina triene	$C_{10}H_{16}$	136.2340	0.7	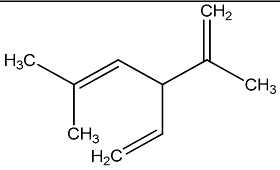	Antimicrobial, anti-inflammatory 1,5,30-32,33,35]
12	11.59	Artemisia alcohol	$C_{10}H_{18}O$	154.25	5.62	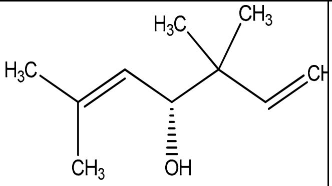	Antimicrobial [24,33,35]
13	12.33	Borneol	$C_{10}H_{18}O$	154.2493	11.12	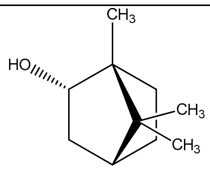	Antioxidant, anti-inflammatory [22,25]
14	13.02	Cirsilineol	$C_{17}H_{14}O_7$	344.0896	2.25	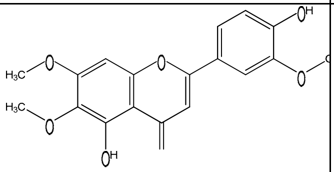	Antioxidant, anticancer, antibacterial, and immunosuppressive

							activity [33,35,43]
15	13.23	Thujone	$C_{10}H_{16}O$	152.2334	0.12	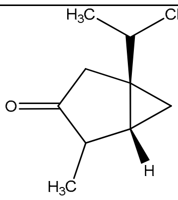	Antioxidant, anti-inflammatory [1,2,5,8,14]
16	13.69	Quercetin	$C_{15}H_{10}O_7$	302.2357	0.39	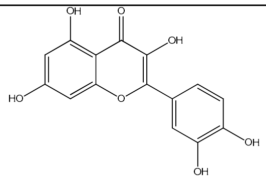	Antioxidant [11,22,25]
17	13.84	Grandisol	$C_{10}H_{18}O$	154.25	28.45	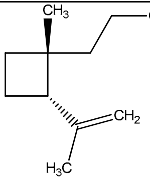	Antimicrobial [1,2,5,8,14]
18	14.65	Naringenin	$C_{15}H_{12}O_5$	272.257	0.53	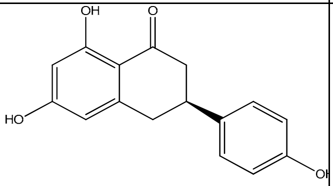	Antidiabetic, gastroprotective, anticancer, antiobesity, immunomodulator, cardioprotective, antimicrobial, nephroprotective [1,2,5,8,11,19]
19	15.53	Artemisia ketone	$C_{10}H_{16}O$	152.233	3.12	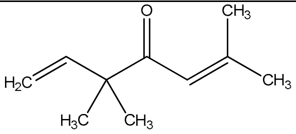	Antimicrobial [24,33,35]
20	16.74	β-caryophyllene	$C_{15}H_{24}$	204.36	5.67	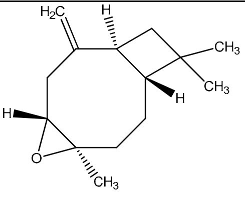	Antibacterial, antioxidant, gastroprotective, anxiolytic, anti-inflammatory [1,2,5,8,14, 24,33,35]

21	17.33	Copaene	$C_{15}H_{24}$	204.36	1.41		Cytotoxicity, antiviral, antibacterial, anti-inflammatory [1,5,8,14,43]
22	17.81	α-amyrin	$C_{30}H_{50}O$	426.7174	0.1		Antinociceptive and anti-inflammatory [30-32]

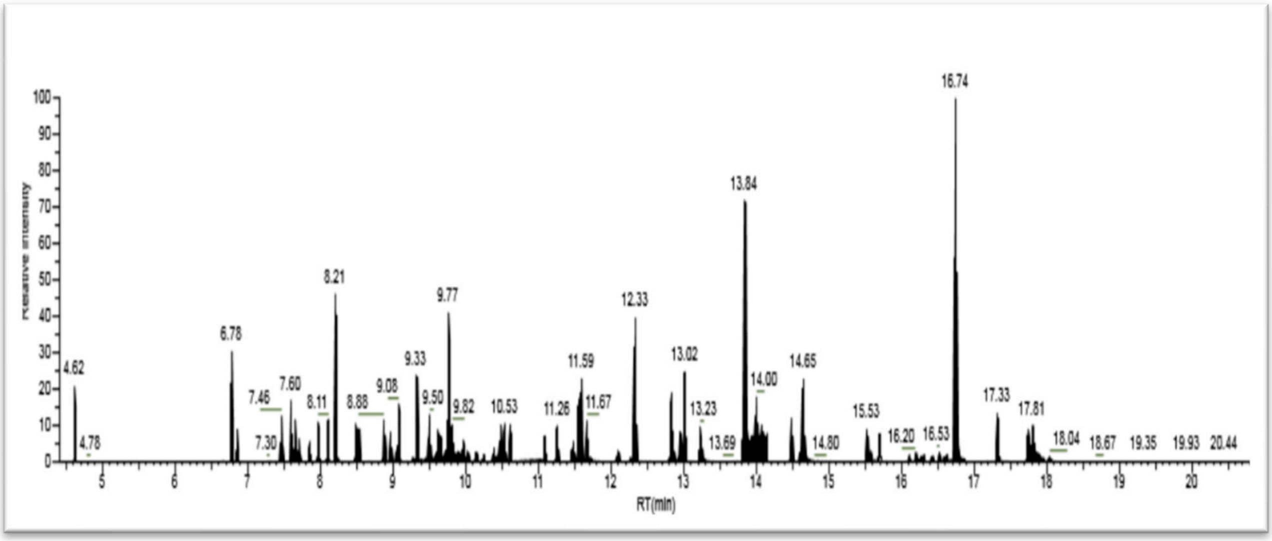

Figure S1. Gas chromatography mass spectrometry of *Artemisia vestita* leaf extract (ALE).
